# Continuous subcutaneous foslevodopa/foscarbidopa infusion for the treatment of motor fluctuations in Parkinson’s disease: Considerations for initiation and maintenance

**DOI:** 10.1016/j.prdoa.2024.100239

**Published:** 2024-02-10

**Authors:** Victor S.C. Fung, Jason Aldred, Martha P. Arroyo, Filip Bergquist, Agnita J.W. Boon, Manon Bouchard, Sarah Bray, Sara Dhanani, Maurizio F. Facheris, Nahome Fisseha, Eric Freire-Alvarez, Robert A. Hauser, Anna Jeong, Jia Jia, Pavnit Kukreja, Michael J. Soileau, Amy M. Spiegel, Saritha Talapala, Arjun Tarakad, Enrique Urrea-Mendoza, Jorge Zamudio, Rajesh Pahwa

**Affiliations:** a Movement Disorders Unit, Westmead Hospital, Westmead, NSW, Australia; b Sydney Medical School, University of Sydney, Sydney, NSW, Australia; c Inland Northwest Research, Spokane, WA, USA; d Selkirk Neurology, Spokane, WA, USA; e Lakeside Dermatology, Libertyville and Gurnee, IL, USA; f Department of Pharmacology, University of Gothenburg, Gothenburg, Sweden; g Department of Neurology, Sahlgrenska University Hospital, Gothenburg, Sweden; h Department of Neurology, Erasmus MC University Medical Center, Rotterdam, the Netherlands; i Clinique Neuro-Lévis, Université Laval, Lévis, QC, Canada; j Centre de Recherche St-Louis, Lévis, QC, Canada; k Banner Sun Health Research Institute, Sun City, AZ, USA; l AbbVie Inc., North Chicago, IL, USA; m AbbVie GK, Tokyo, Japan; n Neurology Department, University General Hospital of Elche, Elche, Spain; o Parkinson’s Disease and Movement Disorders Center, University of South Florida, Tampa, FL, USA; p Texas Movement Disorder Specialists, Georgetown, TX, USA; q Parkinson's Disease Center and Movement Disorders Clinic, Baylor College of Medicine, Houston, TX, USA; r Prisma Health Neurology, Greenville, SC, USA; s School of Medicine, University of South Carolina, Greenville, SC, USA; t Parkinson's Disease and Movement Disorder Center, University of Kansas Medical Center, Kansas City, KS, USA

**Keywords:** Levodopa, Carbidopa, Parkinson’s disease, Motor fluctuations, Subcutaneous infusion, Foslevodopa, Foscarbidopa, Treatment initiation, Treatment maintenance

## Abstract

**Background:**

As Parkinson's disease (PD) advances, management is challenged by an increasingly variable and inconsistent response to oral dopaminergic therapy, requiring special considerations by the provider. Continuous 24 h/day subcutaneous infusion of foslevodopa/foscarbidopa (LDp/CDp) provides steady dopaminergic stimulation that can reduce symptom fluctuation.

**Objective:**

Our aim is to review the initiation, optimization, and maintenance of LDp/CDp therapy, identify possible challenges, and share potential mitigations.

**Methods:**

Review available LDp/CDp clinical trial data for practical considerations regarding the management of patients during LDp/CDp therapy initiation, optimization, and maintenance based on investigator clinical trial experience.

**Results:**

LDp/CDp initiation, optimization, and maintenance can be done without hospitalization in the clinic setting. Continuous 24 h/day LDp/CDp infusion can offer more precise symptom control than oral medications, showing improvements in motor fluctuations during both daytime and nighttime hours. Challenges include infusion-site adverse events for which early detection and prompt management may be required, as well as systemic adverse events (eg, hallucinations) that may require adjustment of the infusion rate or other interventions. A learning curve should be anticipated with initiation of therapy, and expectation setting with patients and care partners is key to successful initiation and maintenance of therapy.

**Conclusion:**

Continuous subcutaneous infusion of LDp/CDp represents a promising therapeutic option for individuals with PD. Individualized dose optimization during both daytime and nighttime hours, coupled with patient education, and early recognition of certain adverse events (plus their appropriate management) are required for the success of this minimally invasive and highly efficacious therapy.

## 1 Introduction

As Parkinson's disease (PD) advances, disabling motor and non-motor symptoms may be experienced due to the combination of progressive nigrostriatal dopaminergic denervation, unreliable absorption of oral levodopa (LD) medications, and the short half-life of oral LD medications in the context of a narrowed therapeutic window [1], [2]. Patients require increasingly complex oral medication regimens, which can be associated with a greater potential for treatment-emergent adverse events (AEs) and lower quality of life, and often necessitate transition to device-assisted therapies.

Foslevodopa/foscarbidopa (also referred to as LDp/CDp or ABBV-951) is a soluble formulation of LD and carbidopa (CD) prodrugs delivered as a 24 h/day continuous subcutaneous infusion (CSCI) for the treatment of motor fluctuations in people with PD [3]. This clinical considerations paper seeks to provide practical recommendations for the successful implementation of LDp/CDp CSCI therapy by reviewing evidence derived from LDp/CDp clinical trial experiences as well as lessons from other subcutaneously administered medications.

### 1.1 LDp/CDp

**Fig. 1:**
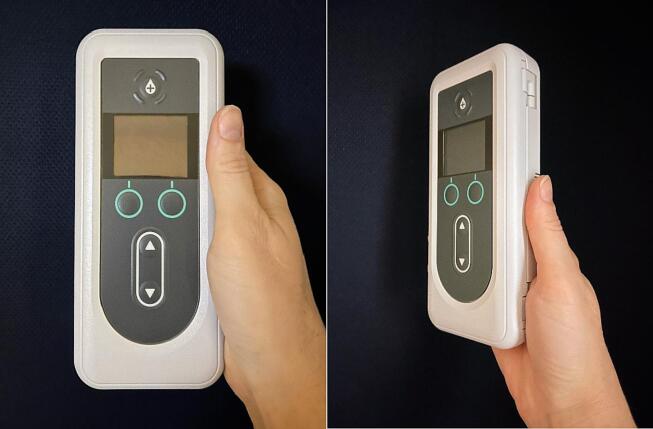
Pump Images.

Upon subcutaneous delivery, LDp and CDp undergo rapid enzymatic conversion via alkaline phosphatases to the pharmacologically active forms LD and CD, respectively [4], [5]. In a Phase 1 healthy volunteer study of LDp/CDp, LD and CD were already detectable in plasma at the first timepoint for which PK samples were collected (30 min after start of infusion) [6]. The drug delivery system consists of: 1) an ambulatory infusion pump (Fig. 1), 2) solution vial containing LDp/CDp, 3) vial adapter, 4) syringe, 5) infusion set including cannula and tubing, 6) carrying accessory, 7) rechargeable batteries, 8) battery charger, and 9) instructions for use (IFUs). The pump can provide infusion rates ranging from 0.15 to 1.04 mL/h [4], in increments of 0.01 mL/h (approximately 1.7 mg of LD equivalents/h), enabling more precise dosing compared to oral LD. The infusion rates allowable by the pump correspond to doses ranging from approximately 600 mg to 4250 mg of LD equivalents/day. Phase 3 trials to date, all registered with www.ClinicalTrials.gov, include one 12-week, double-blind, double-dummy, active-controlled study (NCT04380142) and its open-label extension (NCT04750226), as well as one 52-week open-label safety study (NCT03781167) and its open-label extension (NCT04379050); all conducted in patients with PD experiencing motor fluctuations not controlled by oral therapy. In the 12-week active-controlled study, 24 h/day CSCI of LDp/CDp was compared to oral immediate-release LD/CD (LD/CD-IR). The clinical effects of treatment with LDp/CDp resulted in a statistically significant and clinically meaningful increase in “On” time without troublesome dyskinesia of 2.72 h (versus 0.97 h with oral LD/CD-IR therapy) and an “Off” time reduction of 2.75 h (versus 0.96 h with oral LD/CD-IR therapy) [3]. The 52-week open-label safety study and its open-label extension study provide further clinical evidence of LDp/CDp efficacy [7], [8]. In the open-label safety study, at week 52, normalized “On” time without troublesome dyskinesia increased by 3.8 h and normalized “Off” time decreased by 3.5 h [7]. Further presentation and discussion of efficacy results from the completed 12-week active-controlled study and the completed 52-week open-label safety study have been published elsewhere (see Soileau et al. [3] and Aldred et al. [7]). Additionally, LDp/CDp has generally been safe in both the active-controlled and open-label studies [3], [7], [8], with the majority of AEs reported as non-serious and mild to moderate in severity, and the LDp/CDp systemic safety profile being consistent with the established safety profiles of other LD-containing therapies [9]. Adverse events such as infusion-site events were the leading cause of discontinuations, but these discontinuations generally occurred within the first 4 to 6 weeks of therapy and contributing factors may have included the delivery system learning curve or initial inadequate control of symptoms during the conversion phase (eg, dosing conversion issues). The totality of the data demonstrated that LDp/CDp delivers superior control of motor fluctuations compared with oral LD/CD-IR and offers an effective 24‑hour/day nonsurgical alternative to currently available oral treatments for PD [3], [7], [8]. Both open-label extension trials are currently still in progress. Clinical trials of LDp/CDp cited here are listed in Supplementary Table 1.

All trials in human subjects in the LDp/CDp clinical program were conducted in accordance with the International Council for Harmonisation guidelines, applicable regulations, and the Declaration of Helsinki. All patients provided written informed consent before screening and the Independent Ethics Committee or Institutional Review Board at each study site approved the study protocol, informed consent forms, and recruitment materials before patient enrollment.

## 2 Preparations to enable success with LDp/CDp therapy

PD patients with a wide range of demographic and disease characteristics are suitable candidates for LDp/CDp therapy [3], [7], [10], [11]. A learning curve during the first 10 weeks is to be expected as the patient becomes familiar with the delivery system, and patients should be advised to anticipate a period of titration when the optimal, individualized therapeutic regimen will be determined. Health care providers (HCPs) and resources such as educational materials will play an important role in the education of patients and care partners and are critical to the success of the therapy. Education should focus on adequate preparation for LDp/CDp initiation, setting clear expectations, and building patient confidence. These tools are intended to maximize successful initiation of the therapy and to support better treatment adherence.

### 2.1 Patient selection

In the clinical trials, patients deemed appropriate candidates for LDp/CDp therapy had the following characteristics:

1. Levodopa-responsive PD
2. Recognizable motor fluctuations despite current treatment regimen, with an average daily “Off” time of  ≥ 2.5 h
3. ≥ 400 mg of LD equivalents/day (derived from LD-containing medication and catechol-*O*-methyltransferase [COMT] inhibitors; inclusion criterion in the 12-week, double-blind study only)

Patients with cognitive impairment (Mini-Mental State Examination [MMSE] score < 24) deemed unable to manage the delivery system safely and effectively were not candidates for therapy. In the 52-week open-label safety study, patients with mild cognitive impairment (MMSE score of 19–23, inclusive) were eligible for study enrollment if, in the investigator's opinion, they were able to adhere to all study requirements.

In clinical practice, tools such as MANAGE-PD (www.managepd.com; www.managepd.eu) or screening criteria such as 5-2-1 (5 LD doses/day; OR 2 h of “Off” time; OR 1 h of troublesome dyskinesia) can also be helpful to identify patients potentially eligible for device-aided therapies such as LDp/CDp [12]. While real-world information has not yet been published for LDp/CDp, general considerations for candidate selection have been published for currently approved device-aided therapies. A general consensus for good candidates across all device-aided therapies include patients typically < 70 years of age, with good levodopa response, who may or may not have troublesome dyskinesia, and who still have good cognitive function [13], [14]. Other possible considerations for selection for levodopa infusion therapies may include patients with nighttime disturbances and with limitations in activities of daily living [13]. Presence of mild hallucinations, orthostatic hypotension, and mild cognitive impairment, or the lack of a care partner presence, would potentially warrant HCP discretion [13], [14]. Published literature also advises against the use of device-aided therapies in patients with severe dementia, troublesome hallucinations, and non-transitory psychosis [13].

### 2.2 Care partner considerations

The need for a care partner is highly dependent on individual patient characteristics, and is not necessarily required. In the clinical trials, 51.4 % of patients managed the therapy without the assistance of a dedicated care partner. The need for care partner support or other home assistance may evolve over time; for example, care may be needed during treatment initiation but that need diminishes as the patient gains more experience with the infusion system.

## 3 Best practices for LDp/CDp initiation and optimization

### 3.1 Determining the Base LDp/CDp infusion rate and loading dose

During LDp/CDp initiation, the Base infusion rate is determined by calculating the total dose of LDp/CDp that is required based on a patient’s oral LD-containing medications and COMT inhibitors taken during a 16-hour waking period using a provided conversion algorithm (Table 1). After the patient’s daily LD equivalent dose is determined, a starting hourly Base infusion rate of LDp/CDp is selected from a provided table that accounts for the conversion from a 16-hour dosing period to a 24-hour continuous dosing regimen (Table 2). Rescue LD and concomitant PD medications that do not contain LD (eg, dopamine agonists, monoamine oxidase type B inhibitors, amantadine) are not included in the conversion algorithm, and adjustment of these medications (tapering/discontinuing or changing the dose) are at the discretion of the treating clinician. If HCPs wish to consider conversion of non-LD containing medications, recently updated levodopa equivalent dose (LED) conversion recommendations have been published [15]. If LDp/CDp is initiated in the “Off” state, a loading dose (delivered orally as LD/CD, LD/benserazide, or via pump as LDp/CDp) may be administered to help the patient quickly reach the “On” state (Table 3). This same loading dose may be administered in case of an infusion interruption > 3 h. In the phase 3 clinical trials, the loading dose corresponded to the patient’s first morning dose of LD prior to LDp/CDp initiation.

**Table 1:** Calculating LD Equivalents From LD-Containing Medications.^a^

Medication	Dose Multiplication Factor
Immediate-release LD, including enteral suspension	No adjustment needed (i.e., multiply by 1)
Sustained-release LD, controlled-release or prolonged-release	Multiply by 0.75
Extended-release LD (Rytary®), mg	Multiply by:
0 – 855	0.42
856 – 1755	0.48
1756 – 2340	0.56
≥ 2341	0.67
If any COMT inhibitor is used, multiply sum of calculated LD equivalents from above by 1.33	

LD = levodopa. CD = carbidopa. IR = immediate release. COMT = catechol-*O*-methyltransferase.

Conversion factors provided are based on data from literature [31], [32] The LD dose contained in combined LD/CD/COMT-inhibitor formulations (e.g., Stalevo®) counts as IR and needs to be added to the LD equivalents from all other sources of LD before the sum is multiplied for the COMT-inhibitors correction factor (i.e., do not apply COMT correction factor until all LD equivalents are summed).

a Reprinted from The Lancet Neurology, 21, Michael J Soileau, Jason Aldred, Kumar Budur, Nahome Fisseha, Victor SC Fung, Anna Jeong, Thomas E Kimber, Kevin Klos, Irene Litvan, Daniel O’Neill, Weining Z Robieson, Meredith A Spindler, David G Standaert, Saritha Talapala, Eleni Okeanis Vaou, Hui Zheng, Maurizio F Facheris, Robert A Hauser, Safety and efficacy of continuous subcutaneous foslevodopa-foscarbidopa in patients with advanced Parkinson’s disease: a randomised, double-blind, active-controlled, phase 3 trial, Pages 1099–1109, Copyright (2022), with permission from Elsevier [3].

**Table 2:** Starting Infusion Rate Determination.^a^

Daily LD from LD/CD IR at End of Stabilisation Period (mg/16 h)	Starting Hourly Infusion Rate^b^ (mL/h)
< 500	0.16
500	0.18
600	0.20
700	0.24
800	0.27
900	0.30
1000	0.34
1100	0.37
1200	0.40
1300	0.44
1400	0.47
1500	0.50
1600	0.54
1700	0.57
1800	0.60
1900	0.64
2000	0.67
2100	0.70
2200	0.74
2300	0.78
2400	0.81
2500	0.84
2600	0.88
2700	0.91
2800	0.94
2900	0.98
3000	1.00
≥ 3100	1.04

LD = levodopa. CD = carbidopa. IR = immediate release. LDP = foslevodopa.

a Reprinted from The Lancet Neurology, 21, Michael J Soileau, Jason Aldred, Kumar Budur, Nahome Fisseha, Victor SC Fung, Anna Jeong, Thomas E Kimber, Kevin Klos, Irene Litvan, Daniel O’Neill, Weining Z Robieson, Meredith A Spindler, David G Standaert, Saritha Talapala, Eleni Okeanis Vaou, Hui Zheng, Maurizio F Facheris, Robert A Hauser, Safety and efficacy of continuous subcutaneous foslevodopa-foscarbidopa in patients with advanced Parkinson’s disease: a randomised, double-blind, active-controlled, phase 3 trial, Pages 1099–1109, Copyright (2022), with permission from Elsevier [3].

b Based on LDP concentration of 240 mg/mL and molecular weight conversion factor 100 mg LD = 141 mg LDP. Hourly infusion rate (mL/h) calculated as: [(levodopa equivalents ∙ 0.92 ∙ 1.41) ∕ 240] ∕ X, where X is the number of participant’s awake hours used to determine the levodopa equivalents LE (e.g., X = 16, in the table above).

**Table 3:** Determination of LDp/CDp Volume Recommended for the Loading Dose.

LDp/CDp loading dose volume (mL)	Approximate LED (mg)
0.6	100
0.9–1.2	150–200
1.5–1.8	250–300
2.0	350

CDp, foscarbidopa; LDp, foslevodopa; LED, levodopa equivalent dose, 0.1 mL of LDp/CDp contains 24 mg of foslevodopa (equivalent to approximately 17 mg of levodopa). The pump is capable of delivering a loading dose ranging from 0.1 mL to a maximum of 3.0 mL, in increments of 0.1 mL.

### 3.2 Treatment optimization

#### 3.2.1 Infusion rate adjustments

Like other therapeutic approaches for PD that require individualized optimization, the initial LDp/CDp Base infusion rate will likely require adjustments to achieve optimal symptomatic control. The optimal therapeutic dose of LDp/CDp maximizes “On” time with non-troublesome dyskinesia and minimizes “Off” time. The LDp/CDp infusion rate can be increased or decreased by multiples of 0.01 mL/h (approximately 1.7 mg/h of LD) to fine-tune therapy. One approach based on clinical trial experience is to adjust the infusion rate as a percentage of the Base rate, with ± 10 % as a reasonable starting point for titration. However, some clinical trial investigators noted that many patients only required the smallest adjustment (0.01 mL/h) to optimize symptom control, and that small adjustments may be preferred to avoid extended “Off” periods or sudden dyskinesias.

#### 3.2.2 Alternative infusion rates and extra doses

To facilitate dose optimization and provide flexibility, the pump allows two alternative infusion rates and extra doses to be programmed. A lower alternative infusion rate may be helpful to patients during sleep or nighttime hours and a higher alternative infusion rate may be beneficial when patients feel underdosed during predictable periods of the day (eg, afternoons). In the LDp/CDp clinical program, alternative infusion rates were only allowed in the open-label studies. The median higher and lower alternative infusion rates at the time of the final available prescription were 106.7 % (range 101.2 % to 196.3 %) and 90.5 % (range 39.1 % to 98.7 %) of the prescribed Base infusion rate, respectively.

Enabling the extra dose function on the pump helps patients manage possible acute “Off” symptoms experienced during continuous therapy. The pump allows for the delivery of 17 to 51 mg LD equivalents per extra dose with a minimum of 1 h between extra dose administrations. The delivery of an extra dose via the pump provides a convenient option for patients; however, it is important to note that pharmacokinetic data shows that a dose of LDp/CDp will reach peak LD concentration in approximately 60 to 90 min, compared to 30 to 60 min with oral LD/CD (when administered in a fasted state) [4].

Programming alternative rates and extra doses is optional and at the HCPs discretion; however, the recommendation from the clinical trial investigators is to enable these options during the initiation of therapy, with the low alternative infusion rate being a priority. While the most appropriate nighttime infusion rate for symptom relief and safety has not been systematically studied, overnight infusion rates should seek to improve nighttime and early morning symptoms without eliciting bothersome dyskinesias, hallucinations, or other AEs. Practical experience from clinicians using 24-hour levodopa-carbidopa intestinal gel (LCIG) shows that many patients may require a reduced infusion overnight compared to the Base infusion rate. A review of the 24-hour LCIG literature shows that the overnight infusion rate may be reduced by as much as 50 % to 80 % of the daytime rate [16], [17], [18]. Investigators from the LDp/CDp clinical trials generally suggest initially reducing the overnight infusion rate by 30 % to 50 % of the daytime rate, while they acknowledge that the appropriate overnight infusion rate is highly individualized and may require titration over several weeks. The alternative higher infusion rate may be beneficial during periods of high activity (eg, exercise) or when patients feel underdosed. In the clinical trials, if a patient was using the extra dose feature ≥ 5 times/day, the investigators were advised to consider adjusting the Base and/or alternative rates to address this pattern. If a patient is requiring multiple extra doses on a routine basis, or is primarily using the higher alternative infusion rate, the HCP should consider an infusion rate adjustment either to the Base and/or alternative rates.

#### 3.2.3 General considerations

In the clinical trials, initiation and optimization of LDp/CDp was done almost entirely on an outpatient basis, but depending on the practice setting and country, initiation and optimization may also be done on an inpatient basis. Several factors will dictate how often and at what cadence the patient would need to come to the office for dose adjustment and initial optimization (eg, the patient’s distance from clinic or availability of clinical staff). In the clinical trials, optimization was considered complete when no changes to the infusion rate were made for at least 15 days. The mean number of visits for initial optimization in the phase 3 clinical trials ranged from 2.4 to 3.5 visits, with some participants achieving optimal symptom control in just one visit, and a minority requiring 5 or more visits [3], [7]. At the end of the optimization periods, more participants up-titrated than down-titrated their Base infusion rate; and overall, the mean change from the initial Base infusion rate was small, ranging from 5.5 mg LD/h (8.2 % of the Base infusion rate) to 11.0 mg LD/h (16.0 % of the Base infusion rate).

During the optimization period, it is important to assess the clinical response to the infusion while confirming the correct delivery of the therapy. Delivery challenges such as misplacement or dislodgment of the cannula or pooling of the drug in the subcutaneous space may lead to reduced drug absorption, impacting efficacy and increasing the risk of infusion-site AEs. It is important to note that delivery challenges may occur while the patient/care partner becomes familiar with the delivery system (see “2.2 Care partner considerations” and “4. Navigating the LDp/CDp delivery system and mitigating potential challenges”).

### 3.3 Optimizing concomitant PD medications

During the maintenance period of one open-label phase 3 study, greater than 25 % of patients were able to achieve monotherapy with LDp/CDp after previously being on one or more concomitant PD medications (other than oral LD) [7]. The approach of tapering/discontinuing concomitant PD medications upon optimization of LDp/CDp may be done proactively to help simplify a patient’s treatment regimen or as part of a stepwise management of potential AEs, such as hallucinations or impulse control disorders. In the open-label clinical trials, modifications to concomitant PD medications were allowed during the study and left to the investigator’s judgment. In real-world practice, the decision to taper or discontinue concomitant medications, along with the timing of such changes, is highly dependent on the individual patient profile and must be considered on a case-by-case basis.

## 4 Navigating the LDp/CDp delivery system and mitigating potential challenges

### 4.1 Aseptic technique and proper skin care

Patient/care partner competence in aseptic techniques is essential, given that LDp/CDp is a continuous subcutaneous infusion and patients will be engaged in activities that risk introducing bacterial contamination around the infusion area, such as infusion set application and syringe changes. It is recommended that patients and/or care partners: 1) Wash hands thoroughly with soap and water before preparing the infusion set, 2) Clean the selected infusion site with soap and water followed by an alcohol wipe in an outward spiral motion (vs. back and forth) and wait for alcohol to dry, 3) Clean the medication vial with a separate alcohol wipe, and 4) Avoid touching the tip of any disposable component (eg, syringe tip) [19]. Additional good skin care practices include daily showers (or wipes) to keep the infusion-site area clean, ensuring the infusion set is applied to thoroughly dried skin, trimming (but avoid shaving) if hair removal is required at infusion areas, and closely monitoring skin for irritation potentially caused by skincare products with alcohol or adhesives [20]. Limiting manipulation of the cannula pad is also important, since this can lead to skin irritation, abrasions, and other lesions that may increase the risk for subsequent infection (see Table 4, Table 5 for additional considerations).

**Table 4:** Factors to Consider When Choosing an Infusion Site.

**Body composition and distribution of subcutaneous tissue:** Look for places on the skin where you can “pinch an inch” to ensure there is adequate subcutaneous tissue (eg, for males, more around the abdomen; for females, more around hips and upper thighs). • Avoid skin folds because of difficulty securing the cannula, which could lead to impaired absorption[33] • Avoid areas where edema is present, as it may increase infection risk • Avoid bony areas, or areas near joints, where there is limited subcutaneous tissue and a higher potential for dislodgment • Avoid areas with dry skin or infected/broken skin, as it may increase the risk of infection
**Ability to use alternative infusion site:** It might be difficult for patients to apply the cannula to the flank or to the dominant arm by themselves.
**Areas with excessive hair:** To minimize issues with adhesiveness of cannula pad, avoid areas with excess hair, or consider removing hair without causing blade-related skin trauma. Trimming of hair is preferred. If shaving with a razor, consider doing so 1 or 2 days before cannula insertion. Alternatives to razor shaving are trimming or use of hair removal creams.
**Sweating:** To minimize issues with adhesiveness of cannula pad and risk of folliculitis (especially if blade-shaven), avoid areas where the patient sweats significantly, and avoid tight-fitting clothes on the chosen area. Also consider using commercially available ancillary adhesive materials.
**Sleep position:** Discuss expectations with patient to either adjust sleeping position or consider an alternative infusion site that does not impact sleep habits.
**Physical activity and exercise:** Consider choosing sites with the least risk of being impacted by physical activity, and consider using ancillary adhesive materials to prevent activity-related dislodgement of the cannula and subsequent inadequate infusion delivery (eg, intradermal rather than subcutaneous). Avoid sunscreen or lotion on the area, and if used, ensure the area is cleaned with alcohol pad/wipes and allowed to dry completely before inserting the cannula.
**Patient preference:** Patient’s desire for cannula concealment (thigh and flank) vs. no concealment (arm).

**Table 5:** Recommended Best Practices to Reduce the Risk of Infusion-Site Adverse Events.

Practice aseptic technique when manipulating the infusion delivery system. • Prior to preparing the infusion set for use, wash hands with soap and water • Use a clean workspace when laying out the infusion set components • Clean the vial top with an alcohol wipe prior to puncturing with vial adapter • Clean the infusion site with soap and water prior to use. Then, before placing the cannula, wipe the skin with an alcohol wipe in an outward spiral formation (vs. back and forth) to avoid contaminating the insertion site • Avoid touching the tip of any disposable component (eg, syringe tip) • Allow the area to air dry (approximately 1 min) before placing the cannula
Avoid repenetration. Use a new cannula and select a new site instead.
Change the cannula and the infusion site immediately if unusual discomfort or irritation at the infusion site occurs. In the clinical trials, the infusion set and the infusion site could be left unchanged for up to 3 days (when the drug was infused continuously), but depending on the individual patient circumstances, the cannula and infusion site may require change more frequently than every 3 days. Indications to consider changing the cannula and infusion site more frequently than every 3 days include discomfort, irritation, skin reactions, or signs/symptoms of infusion-site AEs.
When finished with an infusion site, ensure that all drug product has been fully absorbed, and consider massaging the used infusion site to encourage absorption of any remaining drug product from the site. Patients should remember to use proper aseptic techniques while massaging previous infusion site areas.
If concerned about frequent cannula dislodgment issues, consider using commercially available medical tapes/ancillary adhesive materials. Medical tape/adhesives should be placed at least 5 cm from the cannula.
If concerned about infusion-site skin events (particularly cellulitis or abscess), initiate appropriate therapy or refer to an HCP knowledgeable about identification and management of infusion-site skin events in a timely manner.

AEs, adverse events; HCP, healthcare provider

### 4.2 Infusion site selection

The periumbilical area of the abdomen is the preferred infusion site for LDp/CDp due to its ample subcutaneous tissue. We recommend applying the infusion set at least 5 cm (2  in.) away from the navel while also maintaining at least a 2.5-cm (1-in.) distance from the previous infusion site. Cannulas should not be placed in areas of scarred or hardened tissue, stretch marks, skin folds, flexible areas of the body (eg, skin folds, joints near areas of flexion, or bony prominences), or locations where clothing might cause irritation or tug on the cannula (Table 4). A pharmacokinetic study in patients with PD showed that administration of LDp/CDp via the arm, thigh, and flank resulted in nearly equivalent exposure to the abdomen [21]. Long-term safety and efficacy of administration to the arm and thigh have not been evaluated; however, use of alternative infusion sites such as the anterior thighs, posterior arms, and flank are permitted in the phase 3 open-label extension studies.

### 4.3 Cannula management and infusion site rotation

Cannulas are available in 6- and 9-mm lengths. HCPs should consider individual patient characteristics, such as thickness of the abdominal subcutaneous fat tissue, when selecting a cannula length; for example, considering the 9 mm cannula for individuals with higher body mass index (BMI). The appropriate cannula length should be long enough to deliver LDp/CDp to the subcutaneous tissue without infiltrating the muscle wall, which can cause pain and/or occlusion of the cannula. If infusion-site AEs develop, adequate delivery into the subcutaneous space (eg, drug pooling and/or skin nodules, lack of efficacy, frequent cannula dislodgement, infusion-site pain, etc.) should be assessed and cannula length may need to be re-evaluated [22].

An infusion site should be used for a maximum of 3 days [3], [7]. However, the cadence of infusion set changes can be more frequent (eg, every day or every 2 days) and should be determined by the HCP and the individual needs of the patient (Table 5). For example, if infusion-site reactions are observed despite proper aseptic technique, more frequent infusion site changes should be considered. Additionally, some HCPs participating in the phase 3 clinical trials found it helpful to instruct patients to change the infusion site more frequently in the first few weeks following initiation of LDp/CDp, and then extend the interval between infusion site changes only after the patients had mastered the techniques necessary to administer the infusion. The infusion site and infusion set must be changed if the pump is disconnected for > 1 h, or blockage of the cannula may occur.

In case of unsuccessful/poor cannula insertion, a different infusion location at least 5 cm (2 in.) away from the unsuccessful insertion site should be selected for the new cannula, since additional skin trauma at the previous site may increase the risk of infection [19]. A new infusion set should be used for each insertion attempt at a new infusion site. If the cannula is not placed or positioned properly, it can result in discomfort that causes the patient to make frequent manipulations around the cannula, potentially contaminating the area and irritating the infusion site. Patients and care partners should be vigilant about inspecting the infusion site, and if any irritation is noted, the infusion site and infusion set should immediately be replaced to minimize risk of bacterial growth and potential infection [19]. These concepts are not always intuitive for patients, and nonadherence to these good infusion practice protocols have been reported for other continuous subcutaneous therapies [23], [24]. As a result, it is extremely important to provide patients/care partners with this specific information during training on application of the infusion set [25].

### 4.4 Vial and syringe changes

The solution vial contains 10 mL of LDp/CDp solution, with an LDp concentration of 240 mg/mL of solution, for a total of 2400 mg LDp and 120 mg CDp (equivalent to approximately 1700 mg LD and 89 mg CD). This ratio of LDp/CDp was found to be optimal in pharmacokinetic studies [6]. Depending on the total daily dose, the patient may require more than one vial per 24 h. As such, the patient and HCP should discuss the best timing for vial changes, to avoid vial changes during nighttime sleep hours and to accommodate the patient’s daily schedule and routine. Additionally, HCPs may want to advise patients to always carry a spare vial and spare ancillary supplies in case of cannula dislodgement, especially if the patient will be away from home for an extended period of time.

## 5 Adapting to and using the delivery system

Another important aspect of education is to help patients feel confident that they and their care partners can successfully use the delivery system and integrate it into their lifestyle. There is a learning curve associated with the delivery system for the first 10 weeks of use, and during this time, patients and care partners may require additional educational materials, including detailed written instructions, user manuals, instructional videos, hands-on demonstrations, or information on how to contact the manufacturer patient support services between clinic visits. As noted above, home care assistance may facilitate the use of LDp/CDp in specific circumstances or even during specific periods of therapy (eg, during treatment initiation).

### 5.1 Setting expectations

Patient/care partner education about commonly observed AEs and potential risks of LDp/CDp therapy is essential for setting expectations and helps support the patient’s treatment decisions. HCPs should educate patients and care partners on how to reduce the risk of common infusion-site AEs by using proper aseptic technique, good skin care practices, and additional guidance for cannula placement and preventing cannula dislodgement (see below and Table 4, Table 5). HCPs and patients should also share knowledge about how certain concomitant medications (eg, blood thinners) or pre-existing conditions (eg, vasculitis or bleeding disorders) may impact the likelihood of some common infusion-site reactions like bruising and bleeding. While many infusion-site events do not typically require immediate medical attention, it is recommended that clinics and patients have a plan of action if serious reactions needing urgent medical attention occur (especially after hours).

Additionally, HCPs should discuss treatment expectations with the patient prior to initiation of therapy and should advise patients that obtaining maximal treatment benefit will likely require some trial and error, with initial optimization requiring titration of the Base rate, alternative infusion rates, and use of extra dose features.

#### 5.1.1 Daily use and pump interruptions

The pump can be disconnected for brief periods of time up to 1 h (eg, for showering, swimming, etc.). The HCP may want to advise the patient to perform a trial of disconnecting the pump to determine the time for motor symptoms to return. For interruptions > 1 h, a new infusion set (tubing and cannula) must be used and rotated to a different infusion site. If an interruption > 3 h occurs, the patient may re-establish control of motor symptoms through either an oral loading dose, or a loading dose delivered by the pump (assuming the functionality is enabled) before resuming the infusion. Regarding sleep, the clinical trial experience was that with trial and error, patients were able to find comfortable sleeping positions that accommodated the pump without risking cannula dislodgment or pulling the infusion tubing during the night. Practical tips for managing the delivery system are listed in Table 6.

**Table 6:** Recommended Best Practices to Manage the LDp/CDp Infusion System.

• Suggest that patients or care partners record date and time of cannula change, such that the cannula and infusion site are not at risk of being used for greater than 3 days.
• Remind patients that the portable pump carrying accessory can be worn in different ways, and they may need to try a few different configurations before finding the way that works best for them. ‒ Emphasize the importance of finding the best way to position the pump during sleep (eg, placing the pump in the standard carrying accessory, pockets, tucked under clothing, or using a fanny pack) to prevent sleep disruptions.
• Direct patients or care partners to secure the infusion tubing to the abdomen with medical-grade adhesives to avoid infusion site trauma caused by accidental pulling or tugging during exercise or while sleeping.

CDp, foscarbidopa; LDp, foslevodopa

### 5.2 Mitigating cannula dislodgement

Similar to poor cannula insertions, if a cannula becomes dislodged, a new infusion set should immediately be applied in a new location; no attempt to adjust or reseat the dislodged cannula should be made. Cannulas may become dislodged if the infusion tubing is accidentally pulled, or if the adhesion of the infusion set fails (Table 5). These incidents may happen more frequently during physical activity due to movement and/or sweating, and may also occur while sleeping (Table 4). If these incidents occur frequently, using commercially available tapes or adhesives to secure the cannula and/or the infusion tubing closer to the body may be indicated [26].

### 5.3 Managing infusion-site events

Infusion-site events, including local reactions and infections, are characterized by erythema, bleeding, bruising, pain, swelling, and/or tenderness. Such events should be closely monitored, as local reactions and infections may share similar features, but infections require prompt evaluation and treatment (Table 5).

#### 5.3.1 Infusion-site bleeding, bruising, and pain

Recurrent infusion-site bleeding, bruising, or pain should prompt the HCP to evaluate cannula length and whether the cannula is adequately secured. Recurrent bleeding or bruising may indicate an inadequately secured cannula, leading to dislodgement and trauma. If this is the case, the cannula may be secured using additional adhesive, and the patient may consider creating tubing slack and securing the tubing to the body (to avoid tugging of the tubing and resultant cannula dislodgement). If recurrent pain is experienced, the timing of the pain should be investigated. If pain occurs with cannula insertion, it could indicate an inappropriately long cannula may be infiltrating the muscle wall and the cannula length should be adjusted. If the pain is unrelated to cannula insertion, cannula dislodgement should again be considered.

#### 5.3.2 Drug pooling and nodules

While not systematically captured as an AE as defined in the study protocols, anecdotal reports from investigators suggest drug pooling around the infusion site was reported by some LDp/CDp clinical trial patients and is a complication of continuous subcutaneous infusion. Drug pooling may appear as swelling and erythema (with or without drug leakage) at the infusion site. This may be related to cannula removal, dislodgement, inappropriate cannula length, or drug volume. If cannula dislodgement is suspected, the previously outlined recommendations for mitigating cannula dislodgement should be followed. If other reasons for drug pooling are suspected, additional recommendations include rotating the infusion site more frequently (every 1–2 days vs. every 3 days), switching to a longer cannula to avoid intradermal delivery (and subsequent drug pooling), gently massaging or “milking” the infusion site to remove any remaining drug product using aseptic technique (eg, washing hands with soap and water prior to handling the infusion site, using an alcohol wipe if wiping the site), and reducing the infusion flow rate. If drug pooling is present, the infusion set should be changed and applied to a new infusion site.

Subcutaneous skin nodules at the infusion site, or hard areas of subcutaneous tissue, may also develop as a complication of subcutaneous drug delivery and were reported in 24.8 % of patients in the phase 3 clinical trials with LDp/CDp. The majority of skin nodules were nonserious, mild or moderate in severity, and resolved. The median time to onset of the infusion-site nodule was 29 days, and the median duration was 34.5 days. In those patients who experienced infusion-site nodules, the majority (>90 %) continued LDp/CDp therapy. If infusion-site nodules develop during LDp/CDp therapy, the infusion site should be changed immediately and the patient should select a new infusion site away from the nodule, as LDp/CDp infusion near the nodule can be painful and may impact drug absorption [25]. Clinical practice recommendations and observations from the phase 3 clinical trials indicate that local skin massage (eg, using the hand, a spiky rubber massage ball, and/or handheld massage device) may facilitate resolution of an existing nodule [25]. Clinical study sites utilizing local skin massage during the phase 3 clinical trials suggest that patients should routinely incorporate regular massage of previous infusion sites into their daily care regimen if they experience nodules or drug accumulation under the skin. Patients should be taught how to palpate/locate their nodules and to apply firm pressure directly on the area, rotating in a gentle circular motion, for at least 10 min/day. Some patients may experience persistent nodules, and so massaging the area twice a day may be indicated in these cases. Teaching patients to identify nodules is important for selecting an appropriate infusion site location. Additionally, HCPs should perform infusion site assessments during clinic visits.

#### 5.3.3 Managing potential incidents of infusion-site cellulitis and abscess

Infusion-site infections such as infusion-site cellulitis can be a complication of continuous subcutaneous therapy, especially if aseptic technique and good skin care practices are not followed. However, infusion-site infections may occur even in patients who utilize proper aseptic technique [27]. Clues to a diagnosis of cellulitis include spreading erythema, expansion of erythema even after relocation of the cannula and infusion set, and warmth or associated systemic symptoms such as fever. If infusion-site cellulitis is suspected, the HCP should either initiate appropriate therapy or refer the patient for evaluation and treatment. Infusion-site cellulitis was reported in 27.7 % of patients in the LDp/CDp phase 3 clinical trials. In the majority of cases, infusion-site cellulitis was reported based on clinical signs and symptoms; infusion site culture was infrequently performed, and when performed the majority did not identify a bacterial pathogen. The majority of infusion-site cellulitis AEs were nonserious and mild or moderate in severity. Regardless of seriousness or severity, the majority of these events were treated with oral antibiotic treatment. Penicillins and cephalosporins were the most frequently prescribed classes of antibiotics.

A challenge to the diagnosis and treatment of skin events is that inflammatory skin reactions and cellulitis may be difficult to distinguish. Simple inflammatory skin reactions may be due to local skin irritation caused by adhesive material or placement of the catheter in the subcutaneous space. To further complicate diagnosis and treatment, antibiotics prescribed to treat cellulitis (eg, doxycycline) can have both antibacterial and anti-inflammatory properties [28]; thus, a positive response to antibiotic therapy does not necessarily confirm a diagnosis of cellulitis.

Infusion-site abscess can also be a complication of continuous subcutaneous therapy; exam findings may include fluctuance (indicating pus collection within the dermis or subcutaneous space), pain, tenderness, and associated systemic symptoms such as fever. If there is suspicion of infusion-site abscess, timely intervention is key, and the HCP should either initiate appropriate therapy or refer the patient for evaluation and therapy. Infusion-site abscess was reported in 9.8 % of patients in the LDp/CDp phase 3 clinical trials. While the majority of infusion-site abscesses were nonserious and mild or moderate in severity, most of the serious infusion-site abscesses required treatment with oral antibiotics including penicillins, cephalosporins, and sulfonamides. Other treatments included incision and drainage, required in 45.9 % of patients who experienced infusion-site abscess.

### 5.4 Managing systemic events

The non-infusion-site (systemic) AE profile of LDp/CDp was generally consistent with what has been observed with other LD formulations [9]. Most of these events were present during the optimization period, while clinicians determined the most appropriate dose of LDp/CDp, and these events mostly subsided in the maintenance phase of the studies [29], [30].

Hallucinations, especially visual hallucinations, are a common symptom in patients with PD and can be associated with disease progression, comorbid pathologies, and medication. In the open-label phase 3 trials, the incidence of hallucinations ranged from 12.4 % to 17.2 %, which is aligned with rates reported for 24-hour LCIG use [17]. In patients who experience hallucinations following LDp/CDp therapy, a reduction of nighttime infusion of 24-hour LDp/CDp may help avoid the risks of new-onset or worsening hallucinations, psychosis, and nightmares. In the clinical trials, a high proportion of patients who reported hallucinations were using concomitant dopamine agonist therapy. If hallucinations or psychosis are encountered, careful tapering or discontinuation of concomitant medications such as dopamine agonists should be considered by HCPs.

### 5.5 Role of support staff

Experienced nurses and other support staff are valuable patient liaisons who can reinforce education on aseptic technique, good skin care practices, proper infusion site rotation, use of the delivery system, best ways to wear and sleep with the infusion pump, and general troubleshooting.

## 6 Conclusions

LDp/CDp is an individualized continuous subcutaneous therapy for LD-responsive patients with PD whose motor fluctuations are inadequately controlled with oral medication. The features of the infusion pump and its programmable options allow for flexible, personalized infusion rates and dosing that can be adjusted to meet the needs of the patient 24 h/day. LDp/CDp has a favorable benefit-risk profile based on clinical trial experience [3], [7], [8].

During the initiation phase, optimizing dosing and operation of the device requires training and support. Providing patient education while managing expectations can support a successful transition to maintenance therapy. As HCPs become more familiar with LDp/CDp therapy, we anticipate they will be able to titrate LDp/CDp efficiently and will be prepared to address associated potential challenges. Similarly, patients and care partners should anticipate a learning curve as they familiarize themselves with the delivery system and acclimate to 24-hour/day continuous therapy. While the first few weeks after initiation should be highlighted as a transition period, patients and care partners should be reassured that the experience from the clinical trials and the expectation in the clinical setting is that challenges encountered in those first few weeks can often be addressed with training and support. Together, patients and HCPs can optimize the treatment experience with LDp/CDp and maximize its potential benefits on motor symptoms, sleep, and quality of life.

## Role of funding source and acknowledgments

AbbVie and the authors thank all the trial investigators, patients, care providers, and staff who participated in the LDp/CDp clinical trial program. AbbVie Inc. funded the LDp/CDp studies and participated in the design, research, analysis, data collection, interpretation of data, writing, reviewing, and approval of this manuscript. All authors had access to relevant data, participated in the drafting, review, approval, and in the decision to submit this manuscript for publication. No honoraria or payments were made for authorship. Medical writing assistance funded by AbbVie Inc. was provided by: Alicia Salinero, PhD, ISMPP, CMPP, of JB Ashtin; as well as, Sneh Mody, PharmD, MBA, BCCCP, and Matthew R. Distasi, MS, PhD, both of AbbVie Inc. Editorial support funded by AbbVie Inc. was provided by Angela T. Hadsell of AbbVie Inc.

## CRediT authorship contribution statement

**Victor S.C. Fung:** Conceptualization, Investigation, Methodology, Writing – review & editing. **Jason Aldred:** Conceptualization, Investigation, Methodology, Writing – review & editing. **Martha P. Arroyo:** Conceptualization, Investigation, Methodology, Writing – review & editing. **Filip Bergquist:** Conceptualization, Investigation, Methodology, Writing – review & editing. **Agnita J.W. Boon:** Conceptualization, Investigation, Methodology, Writing – review & editing. **Manon Bouchard:** Conceptualization, Investigation, Methodology, Writing – review & editing. **Sarah Bray:** Conceptualization, Investigation, Methodology, Writing – review & editing. **Sara Dhanani:** Conceptualization, Investigation, Methodology, Writing – review & editing. **Maurizio F. Facheris:** Conceptualization, Data curation, Formal analysis, Investigation, Methodology, Writing – original draft, Writing – review & editing. **Nahome Fisseha:** Conceptualization, Data curation, Formal analysis, Investigation, Methodology, Writing – original draft, Writing – review & editing. **Eric Freire-Alvarez:** Conceptualization, Investigation, Methodology, Writing – review & editing. **Robert A. Hauser:** Conceptualization, Investigation, Methodology, Writing – review & editing. **Anna Jeong:** Conceptualization, Data curation, Formal analysis, Investigation, Methodology, Writing – original draft, Writing – review & editing. **Jia Jia:** Conceptualization, Data curation, Formal analysis, Investigation, Methodology, Writing – original draft, Writing – review & editing. **Pavnit Kukreja:** Conceptualization, Data curation, Formal analysis, Investigation, Methodology, Writing – original draft, Writing – review & editing. **Michael J. Soileau:** Conceptualization, Investigation, Methodology, Writing – review & editing. **Amy M. Spiegel:** Conceptualization, Data curation, Formal analysis, Investigation, Methodology, Writing – original draft, Writing – review & editing. **Saritha Talapala:** Conceptualization, Data curation, Formal analysis, Investigation, Methodology, Writing – original draft, Writing – review & editing. **Arjun Tarakad:** Conceptualization, Investigation, Methodology, Writing – review & editing. **Enrique Urrea-Mendoza:** Conceptualization, Investigation, Methodology, Writing – review & editing. **Jorge Zamudio:** Conceptualization, Data curation, Formal analysis, Investigation, Methodology, Writing – original draft, Writing – review & editing. **Rajesh Pahwa:** Conceptualization, Investigation, Methodology, Writing – review & editing.

## Declaration of competing interest

The authors declare the following financial interests/personal relationships which may be considered as potential competing interests: Anna Jeong is an employee of AbbVie Inc., and may hold AbbVie Inc. stock and/or stock options. Agnita J. W. Boon is a study investigator and has served as an advisory board member for AbbVie Inc., Stada, and Ever Pharma. She has received a research grant from Stichting ParkinsonFonds and the Erasmus Trust Fund. Amy M. Spiegel is an employee of AbbVie Inc., and may hold AbbVie Inc. stock and/or stock options. Arjun Tarakad declarations of interest: none. Eric Freire-Alvarez has received advisory, consulting, and lectures fees from AbbVie Inc., Teva, Bial, Zambon, Esteve, UCB, and Neuraxpharm; and is an investigator on studies funded by AbbVie Inc., Neuroderm, Cerevel, Roche, Anavex, Bial, Zambon, Impax, and Irlab. Enrique Urrea-Mendoza has received advisory/consulting fees from: AbbVie Inc., Defeat MSA USA, and Medtronic. Filip Bergquist has received advisory and lecture fees from AbbVie Inc., GKC, and Britannia; and research support in kind from GKC. He is a board member of Arvid Carlsson Research AB and receives salary from the University of Gothenburg and Sahlgrenska University Hospital. Jason Aldred is a member of the faculty of the University of Washington and Washington State University. He is a study investigator and has received honorarium from AbbVie Inc., Allergan (now AbbVie Inc.), Teva, US WorldMeds, Medtronic, and Abbott. He is an investigator in studies sponsored by AbbVie Inc., Biogen, Acadia, Northwestern University, Neuroderm, Massachusetts General Hospital, and AstraZeneca. He is also a scientific advisor for AbbVie Inc. He has a clinical practice through Selkirk Neurology. Jia Jia is an employee of AbbVie Inc., and may hold AbbVie Inc. stock and/or stock options. Jorge Zamudio is an employee of AbbVie Inc., and may hold AbbVie Inc. stock and/or stock options. Manon Bouchard has received honoraria for consultancy, lectures, and advisory boards from AbbVie Inc., Allergan (now AbbVie Inc.), Merz, Ipsen, Lilly, Novartis, Paladin, Sunovion, and UCB. She is an investigator for AbbVie Inc., ES-therapeutics, Biohaven, and Pfizer. Maurizio F. Facheris is an employee of AbbVie Inc., and may hold AbbVie Inc. stock and/or stock options. Michael J. Soileau has received advisory/consulting fees from: AbbVie Inc., Abbott, Mertz Therapeutics, Medtronic, and Supernus. He has received research support from: AbbVie Inc., Cerevel Therapeutics, CND Lifesciences, Praxis Precision Medicine, and Teva. He has served on the speaking bureau for: AbbVie Inc., Amneal Pharmaceuticals, Kyowa Kirin, and Neurocrine Pharmaceuticals. Martha P. Arroyo is a study investigator with Leo Pharma and Allakos, and has received honoraria and consultant fees from AbbVie Inc. and BMS. She has a clinical practice through Lakeside Dermatology. Nahome Fisseha is an employee of AbbVie Inc., and may hold AbbVie Inc. stock and/or stock options. Pavnit Kukreja is an employee of AbbVie Inc., and may hold AbbVie Inc. stock and/or stock options. Robert A. Hauser serves on a scientific advisory board for Inhibikase and Stoparkinson. He has received consulting fees from AbbVie Inc., Amneal Pharmaceuticals, Avanex, Biogen, BlueRock Therapeutics, Forsee Pharmaceuticals, Global Kinetics, Inhibikase, Jazz Pharmaceuticals, Kyowa Kirin, MDCE Suzhou, MedRhythms, Merck, Merz, Neurocrine, Neuroderm, Ovid Therapeutics, PD Neurotechnology, Pharma Two B, Regenxbio, Revance, Sage Therapeutics, Scion NeuroStim, Stoparkinson, Sunovion, Supernus Pharmaceuticals, Tolmar, Tremor Research Group, Tris Pharma, UCB, and Vivifi Biotech. He has received speaking fees from Acorda, Amneal Pharmaceuticals, Cerevel, Inhibikase, Kyowa Kirin, Neurocrine Biosciences, Sunovion, and Supernus. His University has received research support from AbbVie Inc., Aeon Biopharma, Annovis Bio Inc., Artizan Biosciences, Biogen MA, Bukwang Pharmaceutical Co. Ltd., Cavion Inc., Cerevance Inc., Cerevel Therapeutics, Cynapsus Therapeutics, Enterin Inc., Genetech, Global Kinetics Corporation, Hoffman-La Roche Inc., Impax Laboratories, Inhibikase Therapeutics, Integrative Research Laboratories Sweden, Lundbeck Inc., Michael J. Fox Foundation for Parkinson’s Research, National Parkinson’s Foundation, Neuraly Inc., Neurocrine Biosciences, Neuroderm, Parkinson’s & Movement Disorder Alliance, Pharma Two B Ltd., Revance Therapeutics, Sage Therapeutics, Sanofi Pharmaceuticals, Scion NeuroStim, SunPharma, and UCB BioPharma. He holds stock options in Axial Therapeutics, Enterin, and Inhibikase as well as holds stock in Revance Therapeutics. He has received intellectual property interests from a Parkinson’s Disease Diary through his University. He acknowledges a Center of Excellence grant from the Parkinson Foundation. Rajesh Pahwa serves as a consultant for Abbott, AbbVie Inc., ACADIA, Acorda, Adamas, Amneal, CalaHealth, Global Kinetics, Impel, Neuropharma, Kyowa, Lundbeck, Mitsubishi, Neurocrine, Orbis Bioscience, PhotoPharmics, Prilenia, Sunovion, Teva Neuroscience, and US WorldMeds. He receives research support from Abbott, AbbVie Inc., Addex, Biogen, Biohaven, Boston Scientific, EIP, Global Kinetics, Impax, Intec, Lilly, Neuroderm, Neuraly, Parkinson’s Foundation, Pharma 2B, Prelinia, Roche, SIS, Sun Pharma, Sunovion, Theranexus, Theravance, US WorldMeds, and Voyager. He is also an investigator for AbbVie Inc. Sarah Bray has received advisory fees from AbbVie Inc., receives a salary from NSW Health, and is a Nurse Coordinator on studies funded by AbbVie Inc. Sara Dhanani received advisory honoraria from Amneal and AbbVie Inc., and is an investigator on studies funded by AbbVie Inc. Saritha Talapala is an employee of AbbVie Inc., and may hold AbbVie Inc. stock and/or stock options. Victor S. C. Fung receives a salary from NSW Health; has received unrestricted research grants from the Michael J. Fox Foundation, AbbVie Inc., and Merz; and receives royalties from Health Press Ltd. and Taylor and Francis Group LLC.
