# Supplementary material for: Continuous subcutaneous foslevodopa/foscarbidopa infusion for the treatment of motor fluctuations in Parkinson’s disease: Considerations for initiation and maintenance
Source: Clin Park Relat Disord. 2024 Feb 10;10:100239. doi: 10.1016/j.prdoa.2024.100239 (PMC10900117; doi:10.1016/j.prdoa.2024.100239)
Supplement: Supplementary data 1 [file mmc1.docx]

| **Appendix A. Supplementary Table 1. List of Cited LDp/CDp Clinical Trials** | | | |
| --- | --- | --- | --- |
| **Study Title** | **Phase of Development** | **NCT Number** | **Published Results Citation** |
| A single ascending dose study to evaluate the safety, tolerability, and  pharmacokinetics of 24 hour and 72 hour subcutaneous infusions of ABBV-951 in subjects with Parkinson's disease | Phase 1 | NCT03033498 | Rosebraugh et al. *Journal of Parkinson's Disease*. 2021;11(4):1695-1702.[4] |
| A comparative pharmacokinetic study of the bioavailability of ABBV-951 and levodopa-carbidopa intestinal gel plus oral carbidopa-levodopa in healthy volunteers | Phase 1 | NA | Rosebraugh et al. *Parkinsonism & Related Disorders*. 2022;97:68-72.[5] |
| A single dose study to evaluate the safety, tolerability, and pharmacokinetics of ABBV-951 in healthy subjects | Phase 1 | NA | Rosebraugh et al. *Annals of Neurology*.  2021;90(1):52-61.[6] |
| Parkinson's disease: a comparative study of levodopa and carbidopa bioavailability following foslevodopa/foscarbidopa infusion at different subcutaneous sites in Parkinson's disease patients  (Study M20-339) | Phase 1 | NCT05094050 | Results presented (Abstract 62) at the 2023 International Congress of Parkinson's Disease and Movement Disorders, Copenhagen, Denmark, August 27-31, 2023.  Han et al. *Movement Disorders*.  2023;38(Suppl 1):S27.[21] |
| A randomized, double-blind, double-dummy, active-controlled study comparing the efficacy, safety and tolerability of ABBV-951 to oral carbidopa/levodopa in advanced Parkinson's disease patients  (Study M15-736) | Phase 3 | NCT04380142 | Soileau et al. *Lancet Neurology*.  2022;21(12):1099-1109.[3] |
| A 52-Week, open-label, single-arm study to evaluate the safety and tolerability of 24-hour daily exposure of continuous subcutaneous infusion of ABBV-951 in subjects with Parkinson's disease  (Study M15-741) | Phase 3 | NCT03781167  (EudraCT 2018-002144-85) | Aldred et al. *Neurology and Therapy*.  2023;12(6):1937-1958.[7] |
| An open-label extension of Study M15-741 to evaluate the safety and tolerability of 24-hour daily exposure of continuous subcutaneous infusion of ABBV-951 in subjects with Parkinson's disease | Phase 3 | NCT04379050  (EudraCT 2019-004235-23) | Study is ongoing. Interim results presented (Abstract 54) at the 2023 International Congress of Parkinson's Disease and Movement Disorders, Copenhagen, Denmark,  August 27-31, 2023.  Fung et al. *Movement Disorders*.  2023;38(Suppl 1):S23-S24.[8] |
| An open-label extension of studies M15-736 and M20-339 to evaluate the safety and tolerability of 24-hour daily exposure of ABBV-951 in subjects with advanced Parkinson's disease | Phase 3 | NCT04750226 | Study is ongoing. |
| ABBV-951, foslevodopa/foscarbidopa (LDp/CDp); CDp, foscarbidopa; EudraCT, European Union Drug Regulating Authorities Clinical Trials; LDp, foslevodopa; NCT, National Clinical Trial; Suppl, Supplement | | | |
